# Optimizing genomic prediction with transfer learning under a ridge regression framework

**DOI:** 10.1002/tpg2.70049

**Published:** 2025-07-17

**Authors:** Osval A. Montesinos‐López, Eduardo A. Barajas‐Ramirez, Josafhat Salinas‐Ruiz, Abelardo Montesinos‐López, Guillermo Gerard, Paolo Vitale, Susanne Dreisigacker, Carolina Saint Pierre, José Crossa

**Affiliations:** ^1^ Facultad de Telemática Universidad de Colima Colima México; ^2^ Colegio de Postgraduados Campus Córdoba Veracruz Mexico; ^3^ Centro Universitario de Ciencias Exactas e Ingenierías (CUCEI) Universidad de Guadalajara Guadalajara México; ^4^ International Maize and Wheat Improvement Center (CIMMYT) Edo. de México México; ^5^ Colegio de Postgraduados Edo. de México México

## Abstract

Genomic selection (GS) is a predictive plant and animal methodology that allows the selection of plants and animals based on predictions without the need to measure the phenotype. However, its practical application requires challenging prediction accuracy due to the noise observations collected in experiments in these areas. Many strategies and approaches have been proposed to improve the prediction accuracy of this methodology. This paper explores the use of transfer learning in the context of GS. Transfer learning with (1) ridge regression (RR) (Transfer RR) and (2) analytic RR (ARR) (Transfer ARR) were applied from cultivars in the proxy environment to predict those cultivars in the goal environments. Also, we compared the performance of models RR and ARR without transfer learning. We used 11 real multi‐environment datasets (wheat and rice) and evaluated them in terms of Pearson's correlation (Cor) and normalized root mean square error (NRMSE). Our study shows empirical evidence that the Transfer RR or Transfer ARR approaches significantly enhanced predictive performance. Across the datasets, Transfer RR (or Transfer ARR) method improved Cor by 22.962% and NRMSE by 5.757%, in comparison to models RR and ARR. These results underscore the potential of Transfer RR (or Transfer ARR) when enhancing predictive accuracy in this context.

## Core Ideas

- Genomic selection is transforming plant breeding but still faces challenges in achieving high prediction accuracy with limited data.
- Transfer learning adapts models trained on one task to improve performance on a related task, enabling efficient learning with limited data.
- Transfer learning leverages knowledge from a well‐studied domain to enhance prediction in a related, data‐scarce domain.

**Abbreviations**
ARR: analytic ridge regression
Cor: correlation
EYT: elite yield trial
GS: genomic selection
IR: irrigation
NRMSE: normalized root mean square error
RR: ridge regression

## 1 INTRODUCTION

Genomic selection (GS) has revolutionized plant breeding by significantly lowering the expenses associated with phenotyping. This approach leverages machine learning models that are trained on a reference population with both phenotypic and genotypic information. Once trained, these models can predict phenotypic traits for a new population using only genotypic data (Montesinos‐López et al., 2022). As a result, GS is increasingly implemented in breeding programs, allowing for the early identification of promising candidate lines without the need for extensive phenotypic evaluation.

GS has made significant progress in a wide array of crop breeding programs, driving notable improvements (Heffner et al., 2009). In maize, GS has played a critical role in increasing yields, bolstering resistance to diseases, and enhancing drought tolerance (Crossa et al., 2013). For wheat, this technique has led to advancements in grain yield, disease resistance, and quality traits (Crossa et al., 2013; Montesinos‐López et al., 2016). Rice breeding programs have also benefited from GS, which has contributed to better yields, improved resistance to pests and diseases, and greater adaptability to environmental conditions (Bartholomé et al., 2022; Spindel et al., 2015). In soybean, GS has optimized yield, disease resistance, and oil and protein content (Jean et al., 2021; J. Zhang et al., 2016). Barley has seen improved yield, malting quality, and disease resistance (Nielsen et al., 2016), while GS in cassava has boosted yield, disease resistance, and nutritional value (Long et al., 2023; Yonis et al., 2020). Potato breeding has similarly benefited, with increases in yield, tuber quality, and disease resistance (Adams et al., 2023; Habyarimana et al., 2017). These examples highlight GS's widespread impact in advancing essential traits across diverse crops.

However, GS is not yet fully optimized for all plant breeding programs due to several factors that influence its prediction accuracy (Montesinos López et al., 2022). Key challenges include the level of relatedness between training and testing populations, the quality and density of genetic markers, population size and structure, the heritability of the traits being predicted, and the choice of prediction models (Alemu et al., 2024). Additionally, the goals of prediction—whether focusing on tested lines in tested environments, untested lines in tested environments, or even untested lines in untested environments—can also impact accuracy. Factors such as genetic architecture further complicate the achievement of consistent results (Alemu et al., 2024).

To further improve the practical utility of GS, research is continuously aimed at enhancing its predictive accuracy. A diverse range of statistical and machine learning techniques has been investigated, including linear models, mixed models, random forests, support vector machines, Bayesian approaches, and deep learning (Montesinos‐López et al., 2022). Each one of these methods brings distinct advantages, from the simplicity and interpretability of linear and mixed models to the flexibility and power of more complex algorithms such as random forests and deep learning.

Despite these advancements, the inherent noise and complexity in the data collected through plant breeding programs continue to represent significant challenges. The presence of environmental variability, genotype‐by‐environment interactions, and incomplete or imprecise phenotypic data introduces uncertainty that often limits the accuracy of predictions (Crossa et al., 2013). This issue is particularly pronounced when the models are applied to untested lines or environments, where the lack of direct phenotypic information complicates prediction efforts.

As a result, there is a growing need for novel approaches that can better handle these complexities and reduce prediction errors. These may include hybrid models that combine the strengths of multiple techniques, more sophisticated feature selection methods, and advanced algorithms that are better equipped to manage noise and uncertainty in large, complex datasets. Some types of hybrid models are ensembles and transfer learning. Recently authors have demonstrated that combining predictions from different models using simple ensemble approaches results in better prediction accuracy than using any one of the models separately from the others (Kick & Washburn, 2023). Tomura et al. (2023) applied the concept of an ensemble to predict several traits in crop breeding. Authors found that the prediction accuracy in the ensemble approach outperformed other models in most cases. While in the context of genomic predictions Washburn et al. (2021) showed that transfer learning methods can enhance genomic prediction accuracy.

Furthermore, integrating multi‐omics data—such as genomic, transcriptomic, and metabolomic information—into GS models is promising in terms of capturing a more complete picture of trait inheritance and expression. Such innovations are essential to push the boundaries of GS and make it a more reliable and robust tool for plant breeding programs.

Transfer learning is a statistical machine learning technique where a model developed for one task is adapted to a different, but related, task (Pan & Yang, 2010). The key principle behind transfer learning is the reuse of knowledge, in the form of learned features or parameters, from a source domain or dataset to improve learning efficiency and performance in a target domain. This is particularly useful in situations in which the target domain has limited data, since the model can leverage patterns or insights gained from a larger, related dataset (Pan & Yang, 2010).

Transfer learning is often used in scenarios such as natural language processing, image recognition, and regression tasks. The technique typically involves pre‐training a model on a broad dataset, followed by fine‐tuning or adjustment of the model parameters to suit the specific requirements of the target task. By initializing the model with pre‐learned knowledge, transfer learning can reduce training time, enhance model accuracy, and help overcome challenges related to data scarcity or computational constraints (Weiss et al., 2016).

Although transfer learning has been applied in genomic prediction to leverage information from related datasets or populations, its use has not been as extensively explored or rigorously implemented as required to fully harness its potential (Washburn et al., 2020). This limitation highlights the need for more systematic studies and innovative approaches to adapt transfer learning methodologies for genomic prediction tasks effectively (Montesinos‐López, Montesinos‐López, Pérez‐Rodríguez, et al., 2021; Montesinos‐López, Montesinos‐López, Hernandez‐Suarez, et al., 2021).

In regression contexts, transfer learning can involve the transfer of parameter estimates from previous models, which can act as starting points for new models. However, when complete parameter estimates are unavailable or incompatible, techniques like penalized estimation are used to refine these initial estimates, improving the model's performance in the target domain (van Wieringen & Binder, 2020).

Ridge regression (RR) provides a robust framework for genomic prediction and could integrate effectively into transfer learning strategies. Transfer learning leverages information from related datasets or domains to improve performance, especially when training data are limited. This is particularly useful in GS, where environmental or population‐specific data are heterogeneous. The regularization feature of RR prevents overfitting to specific datasets, enabling the development of models that generalize well across domains. For instance, in plant breeding, a model trained on genomic data from one environment can be adapted to predict traits in another environment. Studies like Dar and Baraniuk (2022) and Chen et al. (2015) demonstrate the effectiveness of RR and similar linear models in transfer learning,

Furthermore, the study of Tang and de Sa (2020) addresses the computational challenges of fine‐tuning deep neural networks for transfer learning by leveraging the low‐rank properties of feature vectors produced by these networks. The authors demonstrate success in both supervised and semi‐supervised transfer learning tasks. Moreover, transfer learning with random coefficient RR was analyzed by H. Zhang and Li (2023), which proposes estimators that combine ridge estimates from both target and source models, with weights determined by minimizing empirical estimation or prediction risks. Their approach is applied to predicting polygenic risk scores for lipid traits, demonstrating improved prediction errors compared to single sample or Lasso‐based transfer learning methods.

For the reasons outlined above, this study investigated the potential of transfer learning in GS within a RR framework. This evaluation was conducted using 11 multi‐environment real datasets. In this context, we applied transfer learning by leveraging information from one environment (proxy) to improve predictions in another environment (target). The results of transfer learning were compared to those obtained without applying the transfer process, using cross‐validation to assess predictive performance. The evaluation of the prediction models was conducted using two metrics: the average Pearson's correlation (Cor) and the average normalized root mean square error (NRMSE).

**Core Ideas**

- Genomic selection is transforming plant breeding but still faces challenges in achieving high prediction accuracy with limited data.
- Transfer learning adapts models trained on one task to improve performance on a related task, enabling efficient learning with limited data.
- Transfer learning leverages knowledge from a well‐studied domain to enhance prediction in a related, data‐scarce domain.

## 2 MATERIALS AND METHODS

### 2.1 Datasets

Table 1 outlines the general information of the 11 multi‐environment datasets used in this study. A total of nine datasets are from wheat trials and two belong to rice sets. Also, it is important to point out that the 11 datasets are divided into two subgroups. Subgroup G1 contains all those datasets that share the same lines in all its environments (Prop. shared lines = 1) while subgroup G2 are those datasets that share less than 100% of lines between environments (Prop. shared lines < 1). For this reason, the environments of subgroup G1 are more related than those of subgroup G2. Note that Indica and Japonica datasets pertain to rice, whereas the remaining datasets are related to wheat. Detailed information about the Indica and Japonica datasets can be found in Monteverde et al. (2019).

**TABLE 1:** Description of the datasets.

Dataset number	Dataset (crop and number)	Cultivars (number)	Markers (number)	Environments (number)	Traits (number)	Prop. shared lines	Subgroup
Dataset 1	EYT_1	766	2038	4	4	1	G1
Dataset 2	EYT_2	775	2038	5	4	1	G1
Dataset 3	EYT_3	964	2038	5	4	1	G1
Dataset 4	Indica	327	16,383	3	4	1	G1
Dataset 5	Japonica	320	16,383	5	4	0.510	G2
Dataset 6	Wheat_1	1301	5741	2	1	0.363	G2
Dataset 7	Wheat_2	1403	5741	2	1	0.378	G2
Dataset 8	Wheat_3	1275	5741	2	1	0.347	G2
Dataset 9	Wheat_4	1388	5741	2	1	0.397	G2
Dataset 10	Wheat_5	1398	5741	2	1	0.356	G2
Dataset 11	Wheat_6	1277	5741	2	1	0.096	G2

*Note*: Prop. shared lines denotes the average proportion of lines shared between environments. Subgroup G1 are those datasets that share the same lines (100% sharing) in all environments within a year, whereas in Subgroup G2, the proportion of shared lines is less than 1 (or 100%). For Datasets 1–3 (EYT_1, EYT_2, EYT_3), data are obtained from elite variety trials from CIMMYT from the past wheat breeding program where large trials were established each year under several environments (3–5) under bed planting, irrigated, drought, semi‐drought, late planting, and early planting. Trials in environments within a year combination are the same but different between years. Traits usually collected are days to maturity, days to heading, plant height, and grain yield). Wheat_1 to Wheat_6 are wheat trials from CIMMYT to assess bread wheat quality and it comprises trials across 2 years under irrigated conditions with a varying number of wheat cultivar repeated across years.

#### 2.1.1 Plant materials for Elite Yield Trials (EYTs) and wheat

Data from EYTs conducted by International Maize and Wheat Improvement Center (CIMMYT) are established annually across three to five different environments, encompassing diverse conditions such as varied planting methods, irrigation (IR), drought, semi‐drought, late planting, and early planting. The experimental designs for all trials follow an alpha‐lattice structure with three replicates, and spatial analysis is performed to control for plot‐to‐plot variability in each trial. Traits such as grain yield, heading, lodging, and maturity are typically collected and analyzed. All wheat cultivars included in an EYT (conducted within a single year) are tested across all environments for that year, but none are included in EYTs conducted in subsequent years. EYT includes multiple EYTs, such as EYT_1, EYT_2, and EYT_3 (Juliana et al., 2018).

The datasets Wheat_1 through Wheat_6 represent trials conducted to assess the bread‐making quality of CIMMYT wheat varieties. These trials include some varieties tested under irrigated conditions across different years, with only a subset being repeated between two consecutive years. While the original study by Ibba et al. (2020) analyzed five datasets, this study reorganized some of the cultivars measured across 2 years, expanding the analysis to six datasets. Each dataset focuses on a single trait, with varying numbers of cultivars tested in 2 years (environments).

The statistical analysis of each EYT and wheat is carried out in two stages. In the first stage, the experimental design is accounted for, and spatial analyses are performed to recover inter‐block information and improve capturing plot‐to‐plot variability, respectively. Best linear unbiased estimates (BLUEs) are calculated for each wheat cultivar during this stage.

In the second stage, the BLUEs are used for further analysis, where transfer learning with RR is applied. Similar procedure is applied to Wheat_1 throughout Wheat_6 trials but only under irrigated environment in several years.

### 2.2 RR model

In a general framework, let $x_{i}={\left(x_{i1},\text{…},x_{\mathrm{ip}}\right)}^{T}$, i=1,…,n, represents a covariate vector (marker data in the context of our application). Our objective is to use this information to predict or explain the impact of these variables on a continuous response $y_{i}$ that in our context are traits of phenotypic values. The multiple linear regression model posits a relationship between the covariates and the response is given by the following equation:

(1) $$y_{i}=\beta_{0}+\sum_{j=1}^{p}x_{ij}\beta_{j}+\varepsilon_{i}$$

Here, $\varepsilon_{i}$ represents a random error term with mean 0, *E*($\varepsilon_{i}$) = 0, and is assumed to be independent of $x_{i}$. This error term accounts for measurement errors and the influence of unobserved explanatory variables that may also contribute to explaining the response. The conditional mean of the model is given by $E\;\left(y_{i}|x_{i}\right)=\beta_{0}+\sum_{j=1}^{p}x_{ij}\beta_{j}$, where the conditional distribution of $y_{i}$, given $x_{i}$, is determined solely by the information contained in $x_{i}$.

To estimate the parameters $\beta ={\left(\beta_{0},\beta_{1},\cdots ,\beta_{p}\right)}^{T}\;$, we typically rely on a dataset $\left(x_{i}^{T},y_{i}\right)$, i=1,…,n, often referred to as the training data. In this dataset, $x_{i}$ represents a vector of feature measurements and $y_{i}$ is the corresponding response for the ith individual. When dealing with a large number of features (p) and a small sample size (n), RR is one of the methods most commonly used to estimate β. This method finds the value of β that minimizes the penalized residual sum of squares (Hoerl & Kennard, 1970; Montesinos‐López et al., 2022), defined as follows:

(2) $$\mathrm{PRS}S_{\lambda }\;\left(\beta \right)={\left(y\;-\;X\beta \right)}^{T}\;\left(y\;-\;X\beta \right)+\lambda \sum_{j=1}^{p}\beta_{j}^{2}$$

Here, λ≥0 represents the regularization parameter, which controls the extent to which the beta coefficients shrunk toward zero. When λ=0, the solution for the beta coefficients corresponds to the ordinary least squares (OLS) method. However, as λ increases, the penalized residual sum of squares $\mathrm{PRS}S_{\lambda }\left(\beta \right)\;$ becomes dominated by the penalty term, forcing the penalized solution to shrink toward zero (Christensen, 2011). Generally, when the number of parameters exceeds the number of observations, the OLS estimator becomes invalid. A way to address this issue is by constraining the sum of squares of the beta coefficients, thereby regularizing the estimation process (Wakefield, 2013). When the optimization of Equation (2) was carried out using the library glmnet (Friedman et al., 2010), this method was denoted as the standard RR model, whereas when the optimization of Equation (2) was performed manually (writing our own R code) estimating the beta coefficients as $\hat{\beta }={\left(X^{T}X+\lambda I\right)}^{-1}\;X^{T}y$, this method was called the analytic RR (ARR) model.

### 2.3 Transfer RR methods

In the context of *transfer learning* applied to variety trials in environments, the *goal environments* refer to the specific environmental conditions or sites where improved predictions or insights are desired. For example, if the breeding program focuses on improving performance under *drought‐prone environments*, these drought‐prone conditions would be the *goal* environments, whereas data from *well‐irrigated or semi‐drought environments* might serve as *proxy* environments for transfer learning. The aim is to predict cultivar performance in the *goal* environments with higher accuracy, even if direct trials in these environments are sparse or absent.

To implement the Transfer RR, we assume that we have a proxy dataset $\left(x_{pi}^{T},y_{pi}\right)$, $i=1,\text{…},n_{p}$, and a goal dataset $\left(x_{gi}^{T},y_{gi}\right)$, $i=1,\text{…},n_{g}$. In our context the proxy dataset is the information of an environment that is quite related but different to the goal environment. It is assumed that the same markers were measured in both environments, but of course, due to the nature of both environments, the response variables observed in them are different. Regarding the lines measured, in the proxy environment we assume that for all lines ($n_{p})$ markers and phentotypic values are available, whereas for the goal environments, markers are available for all lines ($n_{g})$, but only a portion of the lines contain phenotypic values ($n_{p}<n_{g}$). This means that for the proxy dataset, all individuals contain phenotypic data and markers, whereas for the goal, only a subset of the whole lines contains phenotypic and markers data. The implementation of the transfer learning model involves the following steps:

Step 1. Using Equation (2) and the complete data of the proxy environments, we compute a ${\hat{\beta }}_{p}$ that denotes the beta coefficients of the proxy data.
Step 2. Then, to the response variable (trait of interest with phenotypic values) of the goal data, we subtract the $X_{g}{\hat{\beta }}_{p}$ and denote this modified response variable as $\;y_{*}=y_{g}\;-X_{g}{\hat{\beta }}_{p}$.
Step 3. Next, we minimize the following penalized residual sum of squares (Hoerl & Kennard, 1970; Montesinos‐López et al., 2022), with the modified response variable obtained in Step 2 as follows:
(3) $$\mathrm{PRS}S_{\lambda }\;\left(\gamma \right)={\left(y_{*}-X_{g}\gamma \right)}^{T}\;\left(y_{*}-X_{g}\gamma \right)+\lambda \sum_{j=1}^{p}\gamma_{j}^{2}$$
Step 4. Next, we compute the transfer learning beta coefficients as ${\hat{\beta }}_{g}={\hat{\beta }}_{p}+\hat{\gamma }.$
Step 5. Finally, for a testing set $X_{\mathrm{tst}}$ from the goal environment, we make predictions using the transfer learning beta coefficients as follows:
$$y_{\mathrm{tst}}=X_{\mathrm{tst}}\;{\hat{\beta }}_{g}$$

When the optimization of Equation (3) was performed with the library glmnet (Friedman et al., 2010), we called this method Transfer RR, whereas when the optimization of Equation (3) was performed manually (writing our own R code) for estimating the gamma coefficients as $\hat{\gamma }={\left(X_{g}^{T}X_{g}+\lambda I\right)}^{-1}\;X_{g}^{T}y_{*}$, this method was named *analytic transfer RR (Transfer ARR)*. The four methods evaluated were implemented in the R statistical software (R Core Team, 2024).

### 2.4 Training the RR, ARR, Transfer RR, and the Transfer ARR models

The proposed transfer learning methods require information of two environments, one served as the goal (target) environment and the other as the proxy environment. All possible combinations of environment pairs were considered to implement the transfer methods (Transfer RR and Transfer ARR), the results for each dataset reflect the performance across all such combinations. During training, conventional methods and transfer learning methods utilized the same training subset of the goal environment, but the transfer methods were enriched with beta coefficients computed from the entire proxy environment during its training process. In contrast, the RR methods were implemented only using the corresponding training subset of the goal environment without the enrichment of its beta coefficients.

#### 2.4.1 Cross‐validation strategy

The study employed a dual cross‐validation approach to evaluate and optimize models.

##### Inner cross‐validation (tuning)

Inner cross‐validation was used within the training data of the outer cross‐validation to optimize the regularization parameter (*λ*). Each outer training set was further divided into an inner training set (80%) and a validation set (20%) using 10 random partitions. Hyperparameters were selected based on average mean square error (MSE) since this is the default in glmnet library. It is important to point out that for selecting the hyperparameter, only the inner training was used for the training process for selecting the optimal hyperparameter.

##### Outer cross‐validation (evaluation)

Outer cross‐validation assessed the genomic prediction accuracy. A 10‐random partition strategy was used, where each partition allocated 80% of the data for training and 20% for testing. Models were trained on for each partition with 80% of the data and the remaining 20% were used as testing set, and this process was done with each of the 10 partitions. Prediction accuracy was reported as the average Cor and NRMSE across the 10 partitions (Montesinos‐López et al., 2022). In the outer cross‐validation the whole training set is used in the training process after the inner training process was used to select the optimal hyperparameter, and this training process used the optimal hyperparameter estimated in the inner training process.

This approach ensured a clear distinction between tuning hyperparameters (inner cross‐validation) and evaluating model performance on independent data (outer cross‐validation). We reported average Cor and NRMSE as performance metrics in the outer cross‐validation because they are widely used in genomic prediction and because they are not scale dependent, and while other metrics are available, these two were considered sufficient and appropriate for the objectives of this study (Montesinos‐López et al., 2022). However, for inner cross‐validations, we used MSE since this is the default metric for glmnet in which the RR and Transfer RR methods were computed using the observed and predicted values.

Note that the size of the training sets used in all models were identical and belong only to a subset of the goal environment. Consequently, both inner and outer cross‐validation processes were conducted under the same conditions. However, when training the transfer models, an adjusted response variable was used instead of the original response variable. This adjusted variable incorporates the beta coefficients computed using the complete data from the proxy environments. For further details, refer to Steps 1 through 5 of the Transfer RR and Transfer ARR models.

### 2.5 Comparing results of genomic prediction accuracy of RR, ARR, Transfer RR, and Transfer ARR

To facilitate the comparisons, we computed the average Cor values of the conventional (RR and ARR) models [AvgCor(RR, ARR)] without the transfer methods and compared them with the average Cor values of the transfer methods [AvgCor(Transfer_RR, Transfer_ARR)] via the relative efficiency computed as $[\frac{\left(\mathrm{AvgCor}\{\mathrm{Transfer}\_\mathrm{RR},\mathrm{Transfer}\_\mathrm{ARR}\}\right)}{\left(\mathrm{AvgCor}\{\mathrm{RR},\;\mathrm{ARR}\}\right)}-1]\times 100$. Similarly, we compared the average NRMSE values of the conventional (RR and ARR) models [AvgNRMSE(RR, ARR)] with the average NRMSE values of the transfer models [AvgNRMSE(Transfer_RR, Transfer_ARR)] via the computation of the relative efficiency as $[\frac{\left(\mathrm{AvgNRMSE}\{\mathrm{RR},\;\mathrm{ARR}\}\right)}{\left(\mathrm{AvgNRMSE}\{\mathrm{Transfer}\_\mathrm{RR},\;\mathrm{Transfer}\_\mathrm{ARR}\}\right)}-1]\times 100$.

## 3 RESULTS

**FIGURE 1:**
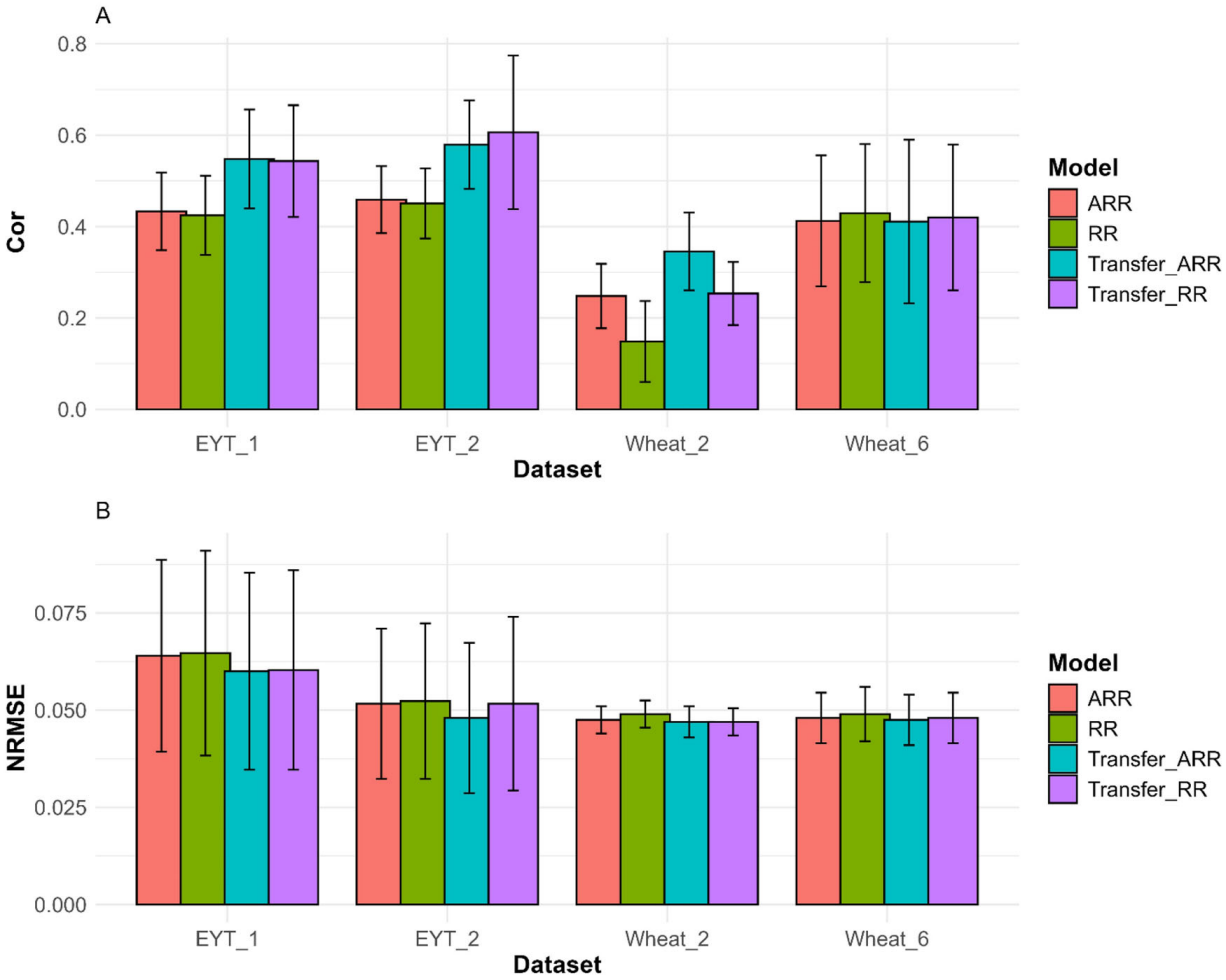
Prediction performance across traits and environments in terms of Pearson's correlation (Cor; A) and normalized root mean square error (NRMSE; B) for the EYT_1, EYT_2, Wheat_2, and Wheat_6 datasets. The models evaluated were ridge regression (RR), analytic ridge regression (ARR), Transfer_RR, and Transfer_ARR.

**FIGURE 2:**
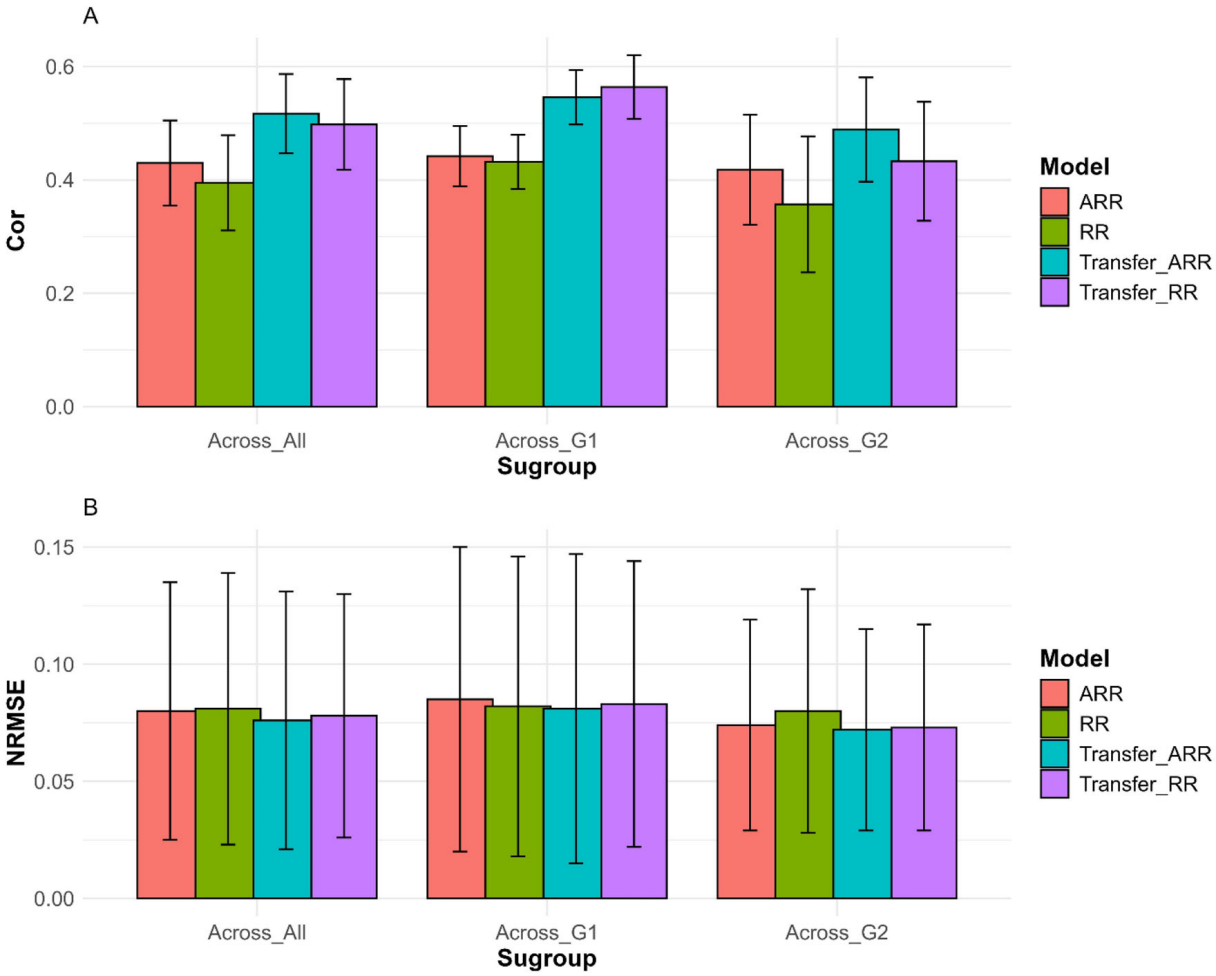
Prediction performance across traits, environments, and datasets in terms of Pearson's correlation (Cor; A) and normalized root mean square error (NRMSE; B) for each model (ridge regression [RR], analytic ridge regression [ARR], Transfer_RR, and Transfer_ARR). The *x*‐axis shows the prediction performance for each subgroup. Across_All are the results across the 11 datasets, Across_G1 are the results across datasets EYT_1, EYT_2, EYT_3, and Indica, and Across_G2 are the results across the remaining datasets that are not included in Across_G1.

**FIGURE A1:**
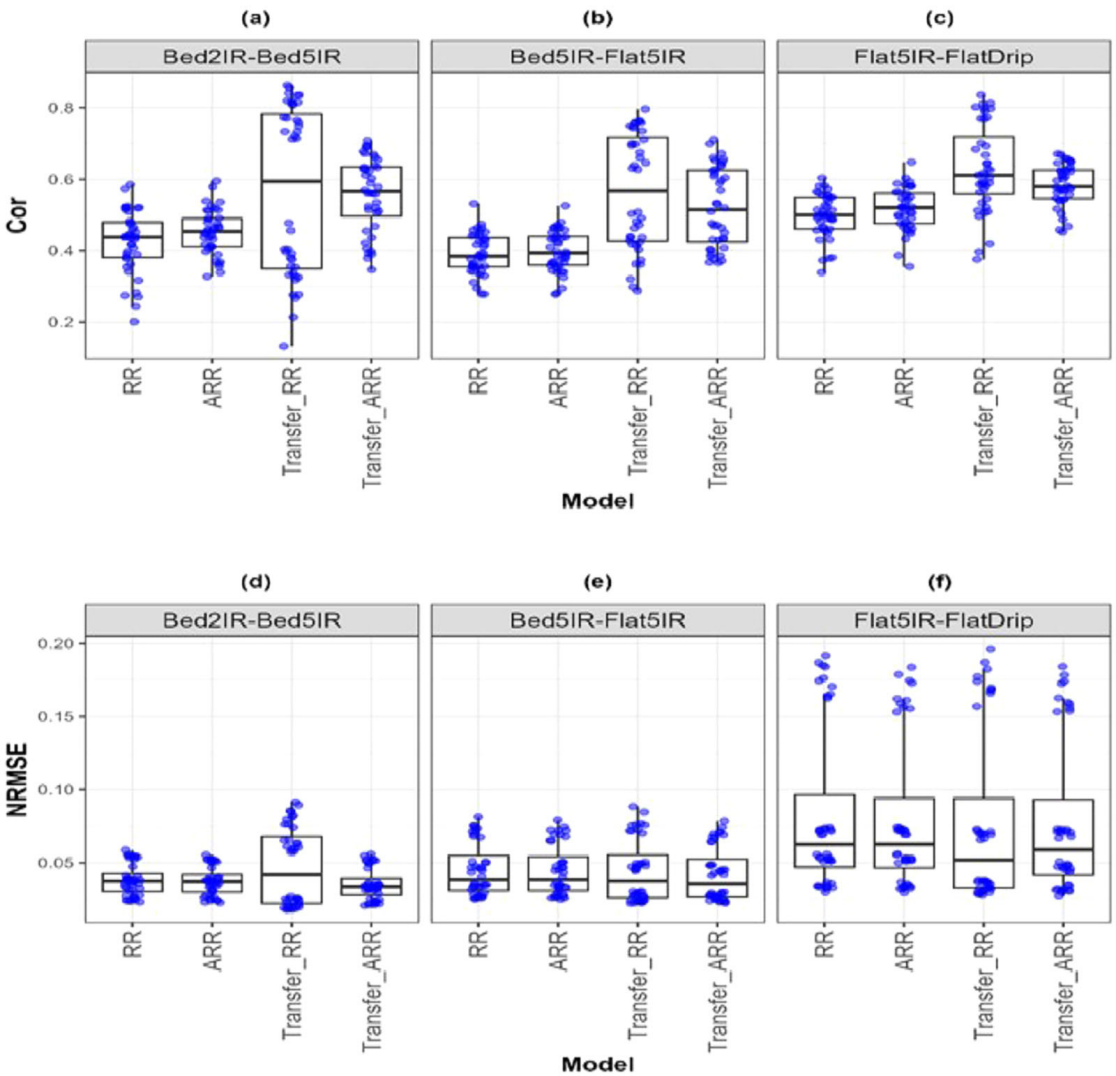
Box plots of Pearson's correlation (Cor) and normalized root mean square error (NRMSE) for the “*EYT_3*″ *dataset* are presented in the top and bottom graphs, respectively. Each blue dot represents a data point per partition and trait. The *x*‐axis shows each model evaluated (ridge regression [RR], analytic ridge regression [ARR], Transfer_RR, and Transfer_ARR). One box plot is presented for each transfer with the label “Env1‐ Env2” to represent the fact that Env1 is the environment used as a proxy and Env2 is the environment used as goal. (a) Box plot correlation of the transfer from environment Bed2IR to Bed5IR. (b) Box plot correlation of the transfer from environment Bed5IR to Flat5IR. (c) Box plot correlation of the transfer from environment Flat5IR to FlatDrip. (d) Box plot NRMSE of the transfer from environment Bed2IR to Bed5IR. (e) Box plot NRMSE of the transfer from environment Bed5IR to Flat5IR. (f) Box plot NRMSE of the transfer from environment Flat5IR to FlatDrip.

**FIGURE A2:**
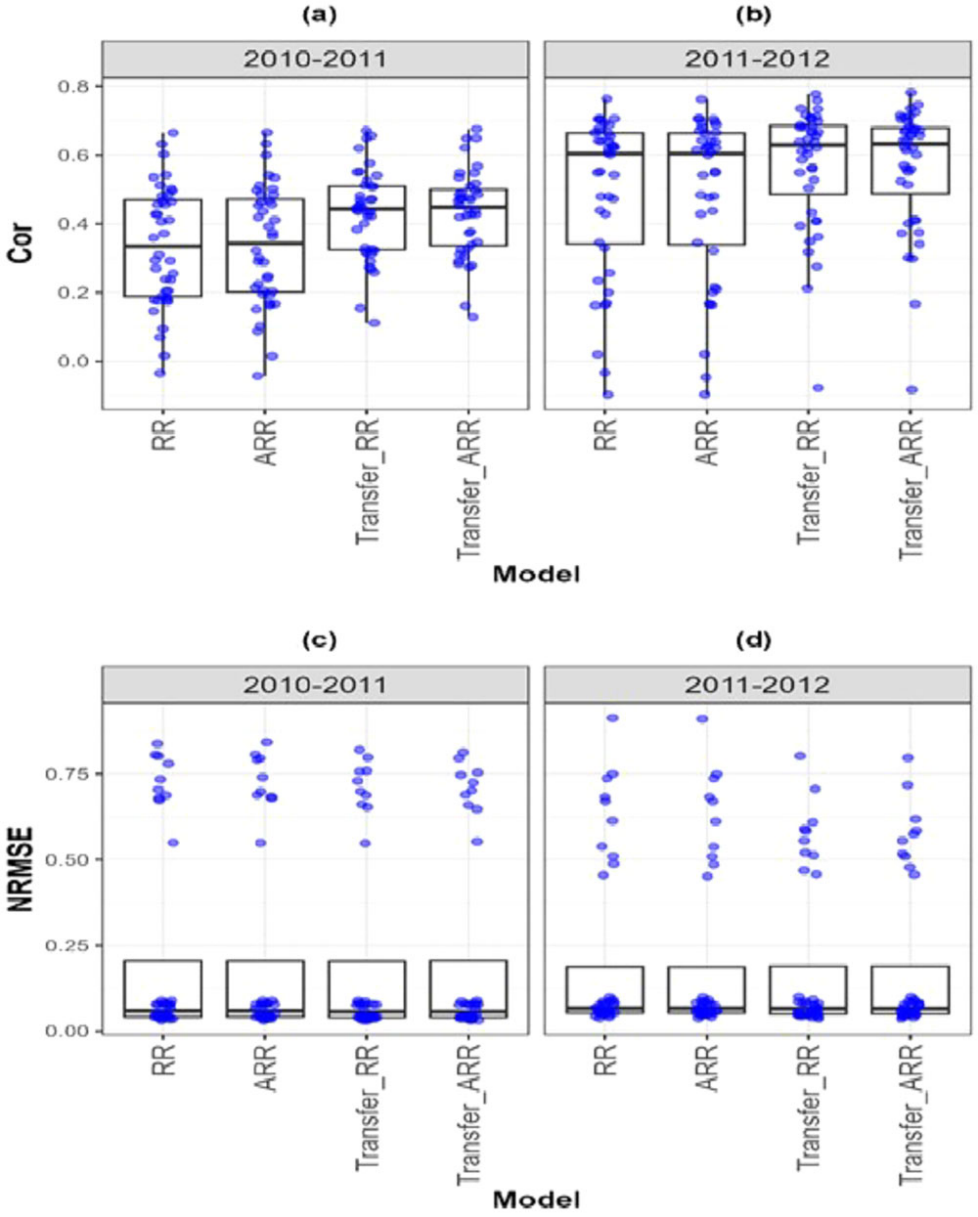
Box plots of Pearson's correlation (Cor) and normalized root mean square error (NRMSE) for the “*Indica*” *dataset* are presented in the top and bottom graphs, respectively. Each blue dot represents a data point per partition and trait. The *x*‐axis shows each model evaluated (ridge regression [RR], analytic ridge regression [ARR], Transfer_RR, and Transfer_ARR). One box plot is presented for each transfer with the label “Env1‐ Env2” to represent the fact that Env1 is the environment used as a proxy and Env2 is the environment used as goal. (a) Box plot correlation of the transfer from environment 2010 to 2011. (b) Box plot correlation of the transfer from environment 2011 to 2012. (c) Box plot NRMSE of the transfer from environment 2010 to 2011. (d) Box plot NRMSE of the transfer from environment 2011 to 2012.

**FIGURE A3:**
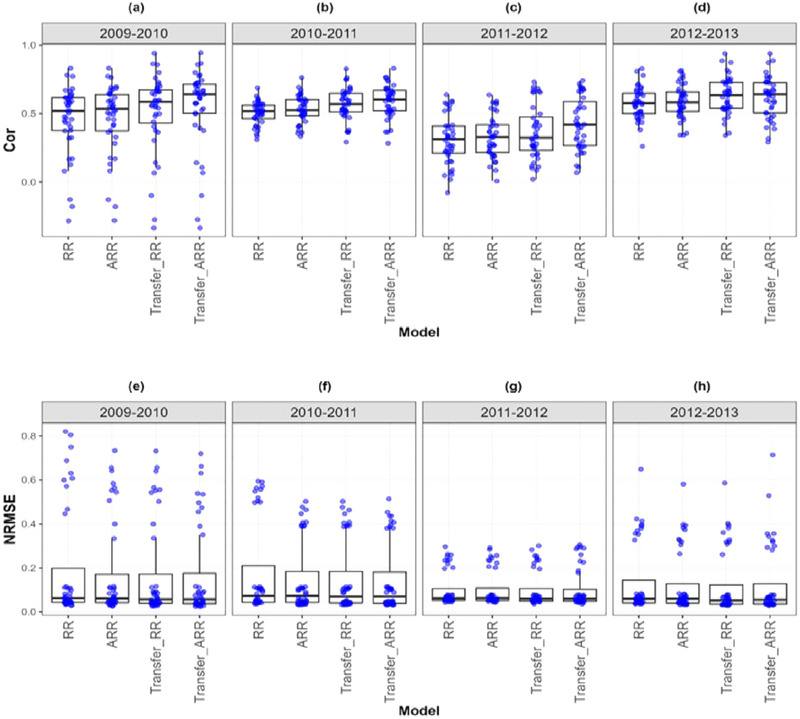
Box plots of Pearson's correlation (Cor) and normalized root mean square error (NRMSE) for the “*Japonica*” *dataset* are presented in the top and bottom graphs, respectively. Each blue dot represents a data point per partition and trait. The *x*‐axis shows each model evaluated (ridge regression [RR], analytic ridge regression [ARR], Transfer_RR, and Transfer_ARR). One box plot is presented for each transfer with the label “Env1‐ Env2” to represent the fact that Env1 is the environment used as proxy and Env2 is the environment used as goal. (a) Box plot correlation of the transfer from environment 2009 to 2010. (b) Box plot correlation of the transfer from environment 2010 to 2011. (c) Box plot correlation of the transfer from environment 2011 to 2012. (d) Box plot correlation of the transfer from environment 2012 to 2013. (e) Box plot NRMSE of the transfer from environment 2009 to 2010. (f) Box plot NRMSE of the transfer from environment 2010 to 2011. (g) Box plot NRMSE of the transfer from environment 2011 to 2012. (h) Box plot NRMSE of the transfer from environment 2012 to 2013.

**FIGURE A4:**
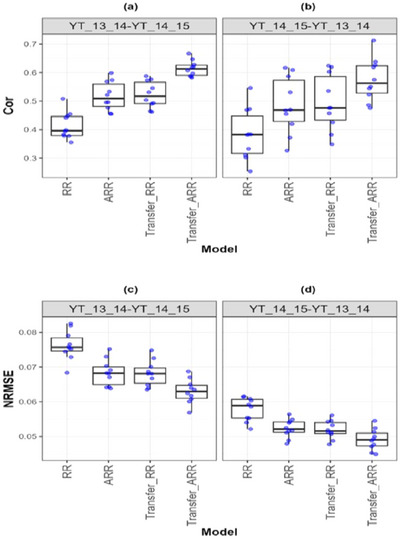
Box plots of Pearson's correlation (Cor) and normalized root mean square error (NRMSE) for the “*Wheat_1*” *dataset* are presented in the top and bottom graphs, respectively. Each blue dot represents a data point per partition and trait. The *x*‐axis shows each model evaluated (ridge regression [RR], analytic ridge regression [ARR], Transfer_RR, and Transfer_ARR). One box plot is presented for each transfer with the label “Env1‐ Env2” to represent the fact that Env1 is the environment used as a proxy and Env2 is the environment used as goal. (a) Box plot correlation of the transfer from environment YT_13_14 to YT_14_15. (b) Box plot correlation of the transfer from environment YT_14_15 to YT_13_14. (c) Box plot NRMSE of the transfer from environment YT_13_14 to YT_14_15. (d) Box plot NRMSE of the transfer from environment YT_14_15 to YT_13_14.

**FIGURE A5:**
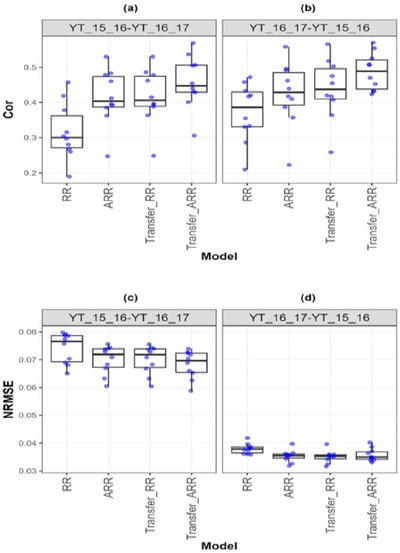
Box plots of Pearson's correlation (Cor) and normalized root mean square error (NRMSE) for the “*Wheat_3*” *dataset* are presented in the top and bottom graphs, respectively. Each blue dot represents a data point per partition and trait. The *x*‐axis shows each model evaluated (ridge regression [RR], analytic ridge regression [ARR], Transfer_RR, and Transfer_ARR). One box plot is presented for each transfer with the label “Env1‐ Env2” to represent the fact that Env1 is the environment used as a proxy and Env2 is the environment used as goal. (a) Box plot correlation of the transfer from environment YT_15_16 to YT_16_17. (b) Box plot correlation of the transfer from environment YT_16_17 to YT_15_16. (c) Box plot NRMSE of the transfer from environment YT_15_16 to YT_16_17. (d) Box plot NRMSE of the transfer from environment YT_16_17 to YT_15_16.

**FIGURE A6:**
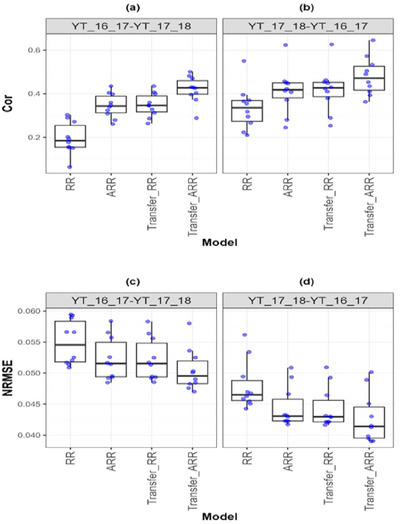
Box plots of Pearson's correlation (Cor) and normalized root mean square error (NRMSE) for the “*Wheat_4*” dataset are presented in the top and bottom graphs, respectively. Each blue dot represents a data point per partition and trait. The *x*‐axis shows each model evaluated (ridge regression [RR], analytic ridge regression [ARR], Transfer_RR, and Transfer_ARR). One box plot is presented for each transfer with the label “Env1‐ Env2” to represent the fact that Env1 is the environment used as a proxy and Env2 is the environment used as goal. (a) Box plot correlation of the transfer from environment YT_16_17 to YT_17_18. (b) Box plot correlation of the transfer from environment YT_17_18 to YT_16_17. (c) Box plot NRMSE of the transfer from environment YT_16_17 to YT_17_18. (d) Box plot NRMSE of the transfer from environment YT_17_18 to YT_16_17.

**FIGURE A7:**
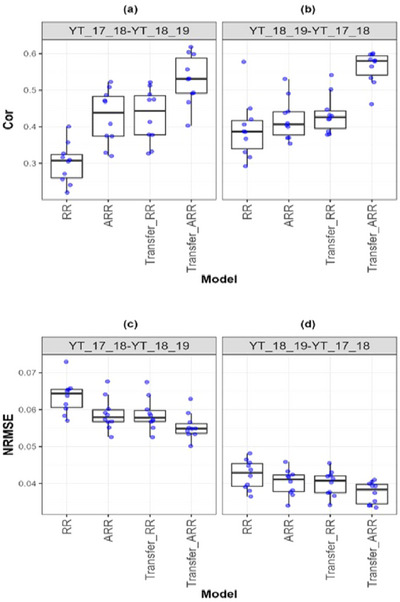
Box plots of Pearson's correlation (Cor) and normalized root mean square error (NRMSE) for the “*Wheat_5*” dataset are presented in the top and bottom graphs, respectively. Each blue dot represents a data point per partition and trait. The *x*‐axis shows each model evaluated (ridge regression [RR], analytic ridge regression [ARR], Transfer_RR, and Transfer_ARR). One box plot is presented for each transfer with the label “Env1‐ Env2” to represent the fact that Env1 is the environment used as a proxy and Env2 is the environment used as goal. (a) Box plot correlation of the transfer from environment YT_17_18 to YT_18_19. (b) Box plot correlation of the transfer from environment YT_18_19 to YT_17_18. (c) Box plot NRMSE of the transfer from environment YT_17_18 to YT_18_19. (d) Box plot NRMSE of the transfer from environment YT_18_19 to YT_17_18.

We divided the results displayed into five sections which contain the results of *EYT_1*, *EYT_2*, *Wheat_2*, *Wheat_6*, *and across all the datasets*, presented in Figures 1 and 2 and Tables 2, 3, 4, 5, 6. The results of the remaining datasets (*EYT_3*, *Japonica*, *Indica*, *Wheat_1*, *Wheat_3*, *Wheat_4*, *and Wheat_5*) are presented in the Appendix, which contains Tables A1, A2, A3, A4, A5, A6, A7 and Figures A1, A2, A3, A4, A5, A6, A7. In each section, we compare the performance of the traditional ridge models (RR and ARR) with the performance of the transfer models (Transfer_RR and Transfer_ARR) in terms of two metrics Cor and NRMSE. The results presented in this section were selected as a representative sample of the results of the datasets, the first two datasets (EYT_1 and EYT_2) with good performance, the third (Wheat_2) with regular performance, and the last one (Wheat_6) with the worst performance. For this reason, we provided the across datasets that offer a general view on the performance of the proposed models across all datasets evaluated.

**TABLE 2:** Prediction performance across traits and environments in terms of normalized root mean square error (NRMSE) and Pearson's correlation (Cor) for the EYT_1 dataset.

Dataset	Model	NRMSE	NRMSE_SD	Cor	Cor_SD
EYT_1	ARR	0.064	0.025	0.433	0.085
EYT_1	RR	0.065	0.026	0.425	0.087
EYT_1	Transfer_ARR	0.060	0.025	0.548	0.108
EYT_1	Transfer_RR	0.060	0.026	0.543	0.122

*Note*: NRMSE_SD denotes the standard deviation of NRMSE and Cor_SD denotes the standard deviation of Cor.

Abbreviations: ARR, analytic ridge regression; RR, ridge regression.

**TABLE 3:** Prediction performance across traits and environments in terms of normalized root mean square error (NRMSE) and Pearson's correlation (Cor) for the EYT_2 dataset.

Dataset	Model	NRMSE	NRMSE_SD	Cor	Cor_SD
EYT_2	ARR	0.052	0.019	0.459	0.073
EYT_2	RR	0.052	0.020	0.451	0.077
EYT_2	Transfer_ARR	0.048	0.019	0.579	0.097
EYT_2	Transfer_RR	0.052	0.022	0.606	0.168

*Note*: NRMSE_SD denotes the standard deviation of NRMSE and Cor_SD denotes the standard deviation of Cor.

Abbreviations: ARR, analytic ridge regression; RR, ridge regression.

**TABLE 4:** Prediction performance across traits and environments in terms of normalized root mean square error (NRMSE) and Pearson's correlation (Cor) for the Wheat_2 dataset.

Dataset	Model	NRMSE	NRMSE_SD	Cor	Cor_SD
Wheat_2	ARR	0.048	0.004	0.248	0.071
Wheat_2	RR	0.049	0.004	0.149	0.089
Wheat_2	Transfer_ARR	0.047	0.004	0.346	0.085
Wheat_2	Transfer_RR	0.047	0.004	0.254	0.069

*Note*: NRMSE_SD denotes the standard deviation of NRMSE and Cor_SD denotes the standard deviation of Cor.

Abbreviations: ARR, analytic ridge regression; RR, ridge regression.

**TABLE 5:** Prediction performance across traits and environments in terms of normalized root mean square error (NRMSE) and Pearson's correlation (Cor) for the Wheat_6 dataset.

Dataset	Model	NRMSE	NRMSE_SD	Cor	Cor_SD
Wheat_6	ARR	0.048	0.007	0.413	0.144
Wheat_6	RR	0.049	0.007	0.430	0.151
Wheat_6	Transfer_ARR	0.048	0.007	0.411	0.179
Wheat_6	Transfer_RR	0.048	0.007	0.420	0.160

*Note*: NRMSE_SD denotes the standard deviation of NRMSE and Cor_SD denotes the standard deviation of Cor.

Abbreviations: ARR, analytic ridge regression; RR, ridge regression.

**TABLE 6:** Prediction performance across traits; environments; and across subgroups G1, G2, and across all datasets for the proposed transfer learning methods and conventional ridge regression (RR) approaches in terms of normalized root mean square error (NRMSE) and Pearson's correlation (Cor) across the 11 datasets.

Model	Subgroup	NRMSE	NRMSE_SD	Cor	Cor_SD
ARR	Across_G1	0.085	0.065	0.442	0.053
ARR	Across_G2	0.074	0.045	0.418	0.097
ARR	Across_All	0.080	0.055	0.430	0.075
RR	Across_G1	0.082	0.064	0.432	0.048
RR	Across_G2	0.080	0.052	0.357	0.120
RR	Across_All	0.081	0.058	0.395	0.084
Transfer_ARR	Across_G1	0.081	0.066	0.546	0.048
Transfer_ARR	Across_G2	0.072	0.043	0.489	0.092
Transfer_ARR	Across_All	0.076	0.055	0.517	0.070
Transfer_RR	Across_G1	0.083	0.061	0.564	0.056
Transfer_RR	Across_G2	0.073	0.044	0.433	0.105
Transfer_RR	Across_All	0.078	0.052	0.498	0.080

*Note*: NRMSE_SD denotes the standard deviation of NRMSE and Cor_SD denotes the standard deviation of Cor.

Abbreviation: ARR, analytic ridge regression.

### 3.1 Dataset EYT_1

Figure 1 illustrates the prediction performance across traits and environments for the EYT_1 dataset, evaluated using the Pearson's Cor and the NRMSE. In terms of Cor, the conventional methods achieved an average value of 0.429, which was lower than the average Cor of the transfer methods (Transfer_RR and Transfer_ARR) at 0.546. This represents a 27.2% improvement in prediction accuracy for the transfer methods (Figure 1). Detailed results for each method are provided in Table 2.

Regarding NRMSE, the conventional methods reported an average value of 0.064, whereas the transfer methods achieved an average value of 0.060, reflecting a 6.9% improvement (Figure 1). Individual results for each method are also detailed in Table 2.

### 3.2 Dataset EYT_2

Figure 1 depicts the predictive performance across traits and environments for the EYT_2 dataset, assessed using the Pearson's Cor coefficient and the NRMSE.

In terms of Cor, the conventional methods yielded an average value of 0.455, which was inferior to the mean Cor of the transfer methods (Transfer_RR and Transfer_ARR) at 0.593. This signifies a 30.341% enhancement in predictive accuracy for the transfer approaches (Figure 1). Comprehensive results for each method are presented in Table 3.

For NRMSE, the methods exhibited an average value of 0.052, whereas the transfer methods achieved a slightly lower average value of 0.050, representing a 4.3% improvement (Figure 1).

### 3.3 Dataset Wheat_2

Regarding Cor, the conventional methods produced an average Cor of 0.198, which was lower than the average Cor of the transfer methods (Transfer_RR and Transfer_ARR) at 0.300. This corresponds to a 51.082% increase in predictive accuracy for the transfer approaches (Figure 1). Detailed results for each method are provided in Table 4.

For NRMSE, the conventional methods reported an average value of 0.048, whereas the transfer methods achieved a marginally lower value of 0.047, indicating a 2.7% improvement (Figure 1). Additional detailed results for each method are available in Table 4.

### 3.4 Dataset Wheat_6

The comparison for this dataset employed a methodology consistent with that applied to previous datasets. In terms of Cor, the conventional methods achieved an average Cor of 0.421, slightly exceeding the average Cor of the transfer methods (Transfer_RR and Transfer_ARR), which was 0.416. This represents a 1.323% decrease in predictive accuracy for the transfer approaches compared to the conventional methods (Figure 1). Results for each method are presented in Table 5.

With respect to NRMSE, the conventional methods yielded an average value of 0.049, while the transfer methods reported a slightly lower value of 0.048, reflecting a 1.6% improvement (Figure 1). Further detailed outcomes for each method can be found in Table 5.

### 3.5 Across data

Across all datasets presented in Figure 2, it is evident that the conventional models (RR and ARR), with an average Cor of 0.413, were outperformed by the transfer models (Transfer_RR and Transfer_ARR), which achieved an average Cor of 0.508, representing a 23.03% improvement (Table 6).

Additionally, as shown in Table 6 and Figure 2, the conventional models reported an average NRMSE of 0.080, which was higher than the average NRMSE of 0.077 achieved by the transfer models, reflecting a 4.5% improvement. Comprehensive individual results for each method are detailed in Table 6.

Table 6 reveals that, in terms of Pearson Cor, subgroup G1, which exhibits the highest degree of relatedness between environments (comprising datasets EYT_1, EYT_2, EYT_3, and Indica), achieved the most substantial improvement, with a 27.00% increase when comparing transfer learning models to conventional methods. In contrast, subgroup G2 demonstrated a more modest gain of 18.960%. Notably, the poorest performance was observed in the Wheat_6 dataset, which has the lowest degree of relatedness among all datasets analyzed.

The results across the datasets demonstrate that the proposed transfer learning methods generally outperform conventional methods. Notably, this pattern of superiority is evident both across datasets and within individual datasets, underscoring the robustness and effectiveness of the proposed method. Despite the difficulty in separating dataset size from the proportion of shared lines—since datasets with more shared lines (G1) were also the smaller datasets, whereas those with fewer shared lines (G2) were the larger datasets—the degree of relatedness between environments emerges as a critical factor. When the relatedness between the proxy and goal environments is negligible, the transfer learning method shows no significant improvement over conventional methods. However, as the relatedness between the proxy and target environments strengthens (genetic similarity, environmental similarity, etc.) the accuracy gains achieved through the transfer learning approaches become increasingly pronounced. These findings highlight the importance of leveraging environmental similarity to maximizing the advantages of transfer learning in genomic predictive modeling.

## 4 DISCUSSION

GS has emerged as a transformative predictive methodology in plant breeding, offering the potential to accelerate genetic gains and improve crop traits (Alemu et al., 2024). Despite its promise, its practical application remains challenging due to the complexity of plant breeding experiments, where numerous environmental and genetic factors are difficult to control and can significantly affect the accuracy of GS predictions, making it harder to implement the method reliably across different contexts. To address this issue, researchers have explored a range of approaches aimed at enhancing the prediction accuracy of GS models. These include integrating advanced statistical techniques, incorporating environmental covariates, and leveraging multi‐trait and multi‐environmental data to improve robustness and reliability in real‐world applications (Alemu et al., 2024).

For this reason, our research focuses on exploring the application of transfer learning to enhance the prediction accuracy of the GS methodology. Transfer learning, which involves leveraging knowledge gained from one context to improve performance in another, has shown great promise in fields such as computer vision and natural language processing (Weiss et al., 2016). Applying this approach to GS allows us to take advantage of models trained on well‐characterized datasets to improve predictions in less studied or more challenging environments. By transferring learned patterns from related crops, traits, or environments, transfer learning can help overcome the limitations posed by small or incomplete datasets, as well as variability in environmental conditions. This strategy not only improves model generalization but also opens new opportunities to streamline breeding programs, especially in regions where experimental data are scarce or highly variable.

In this research, our results across the 11 datasets provide strong empirical evidence that the transfer learning approach significantly improves prediction performance. Specifically, in terms of Cor, the prediction accuracy increased by 23.03% (see Table 6) compared to the conventional methods. Similarly, for the NRMSE, the transfer learning methods achieved a gain of 4.5% over the conventional approaches. These findings underscore the effectiveness of transfer learning in enhancing prediction accuracy, particularly by leveraging additional knowledge from related datasets, which helps mitigate the limitations of conventional methods when faced with complex or variable environments.

However, it is crucial to highlight that the variability observed in the results in the 11 different datasets evaluated using the proposed transfer regression methods is partly attributable to the degree of relatedness between the proxy and goal environments. The proposed methods perform optimally when there is a strong Cor between these environments (as observed in those environments classified as group G1); conversely, a lower degree of relatedness leads to poorer prediction performance (as observed in those environments that were classified as group G2). This represents a significant limitation of the proposed methods, as a lack of relatedness can result in negative transfer as was observed in Wheat_6 dataset. Negative transfer learning occurs when knowledge transferred from a proxy environment adversely affects performance in the goal domain, often due to fundamental differences that lead to inaccurate or misleading information being applied. As a result, predictions may be worse than if no transfer had been utilized at all.

Also, it is important to clarify that both the transfer models and conventional models were trained using the same dataset from the goal environment. However, a key distinction lies in the transfer learning process: The transfer models incorporated beta coefficients learned from proxy environments, which were estimated in a separate, independent training process. Thus, while both models technically rely on the same training set from the goal environment, the transfer models benefit from an additional source of information, the precomputed beta coefficients from the proxy environment. This distinction is crucial when evaluating their performance, as the observed advantage of the transfer models depends not only on the training data itself but also on the structural knowledge transferred from the proxy environment.

Based on these promising results, further exploration of transfer learning in GS is warranted. The substantial gains in prediction accuracy, as proven by improvements in both Cor and NRMSE, highlight the potential of this approach to address key limitations in conventional GS methods. Given that plant breeding often involves working with incomplete or limited datasets, particularly in challenging environments or less‐studied crops, transfer learning provides a means to overcome these barriers by leveraging existing knowledge from related datasets. Additionally, the complexity of genotype × environment interactions require more robust models, capable of generalizing across diverse conditions. Transfer learning not only enhances the accuracy of predictions but also holds the promise of making GS more widely applicable and efficient in real‐world breeding programs. By continuing to explore and refine transfer learning methodologies, we can unlock new possibilities for the acceleration of genetic gains, especially in regions with scarce data or highly variable environmental conditions.

Given that transfer learning has shown significant improvements in prediction accuracy across various fields of science and technology (Pan & Yang, 2010), our application of this approach within the context of GS represents a novel contribution. While this research used a transfer learning model based on a penalized RR framework, we recognize that this is only one potential approach. There is substantial room for further exploration of transfer learning using alternative methods, including Bayesian approaches, advanced machine learning algorithms, and deep learning techniques. Each of these methods could offer unique advantages in terms of model flexibility, the ability to capture complex genotype–environment interactions, and improved generalization across different breeding environments.

### 4.1 Comparing transfer learning methods with alternative machine learning methods

While other methods like neural networks or ensemble approaches might capture complex nonlinear relationships, they often require large datasets and intensive tuning, which may not always be feasible in genomic contexts. Transfer RR strikes a balance by being simpler, faster, and effective for linear relationships. Furthermore, RR's ability to handle high‐dimensional genomic data, its computational efficiency, and its effectiveness in preventing overfitting make it an excellent choice for transfer learning in genomic prediction. It also aligns well with the linear structure of many genomic datasets, ensuring both practical applicability and scientific interpretability.

Moreover, beyond the Transfer RR framework, there is potential to explore more sophisticated strategies to enrich goal (target) environment data with proxy environment information. These could include methods that incorporate environmental and other omics data, multi‐trait data, or ensemble techniques, all of which could enhance the power of GS. Such advancements would help address the inherent limitations of current GS methods, particularly in scenarios involving incomplete or sparse data.

The results of this study demonstrate that RR combined transfer learning provides a robust method of genomic prediction and integration. Some previous studies (Dar & Baraniuk, 2022; Chen et al., 2015; H. Zhang & Li, 2023; Tang & de Sa, 2020) demonstrate the effectiveness of RR and similar linear models in transfer learning.

In practical terms, goal (target) environments might lack sufficient direct experimental data, making it essential to use data from other environments (proxy environments) to make informed predictions. These goal environments are critical for evaluating the performance of wheat cultivars because they might align with the target population of environments (TPE) for which new varieties are being developed using a sparse testing scheme where not all the environments in the TPE (as well as the cultivars) were sampled or not all varieties were sampled in all of the environments in the TPE. Goal environments often represent the regions or conditions or TPE where breeders aim to introduce improved varieties to maximize yield, stress tolerance, or other desired traits.

### 4.2 Discussion of appendix results

The results presented in the Appendix strongly reinforce the utility of transfer learning in genomic prediction across both crop types and environmental conditions. Notably, in the EYT_3 dataset (Table A1; Figure A1), which involves similar IR systems (Flat5IR→FlatDrip), Transfer_ARR significantly improved the Cor (from 0.517 to 0.629) and slightly reduced NRMSE (from 0.081 to 0.078). This reflects the high effectiveness of knowledge transfer under similar agronomic management practices, especially within the same breeding cycle.

In Indica rice (Table A2; Figure A2), a transfer from 2011 to 2012 increased Cor from 0.487 (ARR) to 0.564 (Transfer_ARR) and reduced NRMSE from 0.205 to 0.191. Despite inter‐annual variability, the consistency of genetic evaluation environments enabled successful transfer. Similarly, in Japonica rice (Table A3; Figure A3), though the Cor gain was more modest (from 0.588 to 0.619), the maintenance of NRMSE at 0.131 suggests that while the signal strength may saturate, transfer learning still stabilizes prediction performance across years.

In the wheat datasets, similar patterns emerge. For Wheat_1 (Table A4; Figure A4), Transfer_ARR yielded a substantial Cor increase (from 0.518 to 0.614) and NRMSE reduction (from 0.068 to 0.063), affirming transfer learning's benefits in irrigated wheat systems. Wheat_3 (Table A5; Figure A5) showed moderate yet consistent improvement (Cor from 0.415 to 0.458; NRMSE from 0.070 to 0.068), even under potentially less correlated environmental conditions. In Wheat_4 (Table A6; Figure A6), the Cor increase from 0.412 to 0.480 with a corresponding NRMSE drop (0.044 to 0.043) supports the idea that transfer learning remains advantageous even when environments are only moderately related.

The most striking result came from Wheat_5 (Table A7; Figure A7), where Transfer_ARR outperformed ARR with a Cor gain of 0.135 and an NRMSE reduction from 0.059 to 0.055. This scenario likely represents an optimal case where the proxy and target environments are strongly aligned in terms of genetic, agronomic, and climatic conditions, allowing Transfer_ARR to capture consistent genotype performance patterns more effectively.

Overall, these findings support several conclusions:

1. Transfer learning offers consistent improvement across crops (wheat, Indica, Japonica rice) and years.
2. Gains in predictive accuracy were observed not only for yield but also for other traits like protein content.
3. The Transfer_ARR method generally outperformed Transfer_RR, likely due to improved numerical stability, especially when data dimensionality is high or when marker matrices have collinearity.

These results align with recent studies, demonstrating the benefit of model reuse or adaptation across time, location, or management systems in GS. Importantly, the variability in improvement magnitude across datasets also reflects the importance of environmental relatedness, suggesting that transfer learning works best when the proxy and target conditions share similar environmental signal profiles. This study contributes empirical support for including transfer learning as a regular practice in GS pipelines, particularly when phenotypic data collection is resource limited.

## 5 CONCLUSIONS

In this research, we applied transfer learning within the context of GS. Specifically, we transferred knowledge learned from a proxy environment to improve predictions in a target (goal) environment when limited information exist. This transfer process involved learning the beta coefficients from the proxy environment using a RR framework, which were then used to enrich the incomplete information in the goal environment. Our results prove that transfer learning has a significant potential to enhance the prediction accuracy of the GS methodology in goal environments. Across the 11 datasets evaluated, we observed a 22.962% improvement in Pearson's Cor and a 5.757% improvement in NRMSE in comparison to traditional methods. Despite these promising results, we advocate for further research to fully explore the potential of transfer learning in plant breeding, as it holds great promise in terms of addressing the challenges posed by limited or incomplete data in GS applications.

## AUTHOR CONTRIBUTIONS

**Osval A. Montesinos‐López**: Conceptualization; data curation; formal analysis; investigation; methodology; writing—original draft; writing—review and editing. **Eduardo A. Barajas‐Ramírez**: Validation; writing—original draft; writing—review and editing. **Josafhat Salinas‐Ruiz**: Data curation; validation; writing—original draft; writing—review and editing. **Abelardo Montesinos‐López**: Conceptualization; data curation; formal analysis; investigation; methodology; software; writing—original draft; writing—review and editing. **Guillermo Gerard**: Methodology; writing—original draft; writing—review and editing. **Paolo Vitale**: Conceptualization; investigation; writing—original draft; writing—review and editing. **Susanne Dreisigacker**: Investigation; methodology; writing—original draft; writing—review and editing. **Carolina Saint Pierre**: Conceptualization; funding acquisition; writing—original draft; writing—review and editing. **José Crossa**: Investigation; methodology; writing—original draft; writing—review and editing.

## CONFLICT OF INTEREST STATEMENT

The authors declare no conflicts of interest.

6

## Data Availability

The link https://github.com/alexa11235/TransferOnGenoData contains the phenotypic and genomic data of the 11 datasets used in this study plus the codes for the transfer learning.

## 1

**TABLE A1:** Prediction performance in terms of normalized root mean square error (NRMSE) and Pearson's correlation (Cor) for the EYT_3 dataset.

Dataset	Model	Transfer	NRMSE	NRMSE_SD	Cor	Cor_SD
EYT_3	RR	Bed2IR‐Bed5IR	0.038	0.011	0.424	0.089
EYT_3	ARR	Bed2IR‐Bed5IR	0.037	0.010	0.452	0.063
EYT_3	Transfer_RR	Bed2IR‐Bed5IR	0.047	0.027	0.563	0.235
EYT_3	Transfer_ARR	Bed2IR‐Bed5IR	0.035	0.011	0.557	0.103
EYT_3	RR	Bed5IR‐Flat5IR	0.045	0.018	0.391	0.057
EYT_3	ARR	Bed5IR‐Flat5IR	0.045	0.017	0.398	0.058
EYT_3	Transfer_RR	Bed5IR‐Flat5IR	0.044	0.021	0.564	0.161
EYT_3	Transfer_ARR	Bed5IR‐Flat5IR	0.042	0.018	0.52	0.111
EYT_3	RR	Flat5IR‐FlatDrip	0.084	0.056	0.497	0.062
EYT_3	ARR	Flat5IR‐FlatDrip	0.081	0.052	0.517	0.060
EYT_3	Transfer_RR	Flat5IR‐FlatDrip	0.078	0.059	0.629	0.121
EYT_3	RR	Bed2IR‐Bed5IR	0.038	0.011	0.424	0.089

*Note*: NRMSE_SD denotes the standard deviation of NRMSE and Cor_SD denotes the standard deviation of Cor. In the transfer column, the notation “Env1‐Env2” denotes that Env1 is the environment used as a proxy and Env2 is the environment used as goal.

Abbreviations: ARR, analytic ridge regression; RR, ridge regression.

**TABLE A2:** Prediction performance in terms of normalized root mean square error (NRMSE) and Pearson's correlation (Cor) for the Indica dataset.

Dataset	Model	Transfer	NRMSE	NRMSE_SD	Cor	Cor_SD
Indica	RR	2010–2011	0.223	0.297	0.335	0.175
Indica	ARR	2010–2011	0.223	0.298	0.336	0.174
Indica	Transfer_RR	2010–2011	0.217	0.292	0.431	0.129
Indica	Transfer_ARR	2010–2011	0.217	0.290	0.432	0.125
Indica	RR	2011–2012	0.206	0.261	0.490	0.231
Indica	ARR	2011–2012	0.205	0.260	0.487	0.233
Indica	Transfer_RR	2011–2012	0.191	0.234	0.567	0.178
Indica	Transfer_ARR	2011–2012	0.191	0.234	0.564	0.181

*Note*: NRMSE_SD denotes the standard deviation of NRMSE and Cor_SD denotes the standard deviation of Cor. In the transfer column, the notation “Env1‐Env2” denotes that Env1 is the environment used as a proxy and Env2 is the environment used as goal.

Abbreviations: ARR, analytic ridge regression; RR, ridge regression.

**TABLE A3:** Prediction performance in terms of normalized root mean square error (NRMSE) and Pearson's correlation (Cor) for the Japonica dataset.

Dataset	Model	Transfer	NRMSE	NRMSE_SD	Cor	Cor_SD
Japonica	RR	2009–2010	0.205	0.262	0.455	0.255
Japonica	ARR	2009–2010	0.182	0.225	0.463	0.258
Japonica	Transfer_RR	2009–2010	0.178	0.225	0.510	0.288
Japonica	Transfer_ARR	2009–2010	0.171	0.216	0.544	0.294
Japonica	RR	2010–2011	0.185	0.212	0.504	0.084
Japonica	ARR	2010–2011	0.155	0.163	0.535	0.100
Japonica	Transfer_RR	2010–2011	0.153	0.164	0.569	0.118
Japonica	Transfer_ARR	2010–2011	0.151	0.161	0.590	0.119
Japonica	RR	2011–2012	0.104	0.081	0.308	0.177
Japonica	ARR	2011–2012	0.105	0.082	0.330	0.155
Japonica	Transfer_RR	2011–2012	0.104	0.084	0.364	0.197
Japonica	Transfer_ARR	2011–2012	0.107	0.091	0.427	0.191
Japonica	RR	2012–2013	0.141	0.161	0.577	0.116
Japonica	ARR	2012–2013	0.131	0.145	0.588	0.121
Japonica	Transfer_RR	2012–2013	0.127	0.147	0.632	0.137
Japonica	Transfer_ARR	2012–2013	0.131	0.161	0.619	0.151

*Note*: NRMSE_SD denotes the standard deviation of NRMSE and Cor_SD denotes the standard deviation of Cor. In the transfer column, the notation “Env1‐Env2” denotes that Env1 is the environment used as a proxy and Env2 is the environment used as goal.

Abbreviations: ARR, analytic ridge regression; RR, ridge regression.

**TABLE A4:** Prediction performance in terms of normalized root mean square error (NRMSE) and Pearson's correlation (Cor) for the Wheat_1 dataset.

Dataset	Model	Transfer	NRMSE	NRMSE_SD	Cor	Cor_SD
Wheat_1	RR	YT_13_14‐YT_14_15	0.076	0.004	0.414	0.047
Wheat_1	ARR	YT_13_14‐YT_14_15	0.068	0.004	0.518	0.050
Wheat_1	Transfer_RR	YT_13_14‐YT_14_15	0.068	0.004	0.523	0.047
Wheat_1	Transfer_ARR	YT_13_14‐YT_14_15	0.063	0.003	0.614	0.028
Wheat_1	RR	YT_14_15‐YT_13_14	0.058	0.003	0.384	0.090
Wheat_1	ARR	YT_14_15‐YT_13_14	0.052	0.003	0.486	0.099
Wheat_1	Transfer_RR	YT_14_15‐YT_13_14	0.052	0.003	0.495	0.098
Wheat_1	Transfer_ARR	YT_14_15‐YT_13_14	0.049	0.003	0.575	0.074

*Note*: NRMSE_SD denotes the standard deviation of NRMSE and Cor_SD denotes the standard deviation of Cor. In the transfer column, the notation “Env1‐Env2” denotes that Env1 is the environment used as a proxy and Env2 is the environment used as goal.

Abbreviations: ARR, analytic ridge regression; RR, ridge regression.

**TABLE A5:** Prediction performance in terms of normalized root mean square error (NRMSE) and Pearson's correlation (Cor) for the Wheat_3 dataset.

Dataset	Model	Transfer	NRMSE	NRMSE_SD	Cor	Cor_SD
Wheat_3	RR	YT_15_16‐YT_16_17	0.074	0.005	0.317	0.080
Wheat_3	ARR	YT_15_16‐YT_16_17	0.070	0.005	0.415	0.079
Wheat_3	Transfer_RR	YT_15_16‐YT_16_17	0.070	0.005	0.416	0.079
Wheat_3	Transfer_ARR	YT_15_16‐YT_16_17	0.068	0.005	0.458	0.075
Wheat_3	RR	YT_16_17‐YT_15_16	0.038	0.002	0.372	0.083
Wheat_3	ARR	YT_16_17‐YT_15_16	0.035	0.002	0.424	0.092
Wheat_3	Transfer_RR	YT_16_17‐YT_15_16	0.035	0.002	0.438	0.087
Wheat_3	Transfer_ARR	YT_16_17‐YT_15_16	0.036	0.002	0.487	0.053

*Note*: NRMSE_SD denotes the standard deviation of NRMSE and Cor_SD denotes the standard deviation of Cor. In the transfer column, the notation “Env1‐Env2” denotes that Env1 is the environment used as a proxy and Env2 is the environment used as goal.

Abbreviations: ARR, analytic ridge regression; RR, ridge regression.

**TABLE A6:** Prediction performance in terms of normalized root mean square error (NRMSE) and Pearson's correlation (Cor) for the Wheat_4 dataset.

Dataset	Model	Transfer	NRMSE	NRMSE_SD	Cor	Cor_SD
Wheat_4	RR	YT_16_17‐YT_17_18	0.055	0.004	0.196	0.074
Wheat_4	ARR	YT_16_17‐YT_17_18	0.052	0.003	0.345	0.056
Wheat_4	Transfer_RR	YT_16_17‐YT_17_18	0.052	0.003	0.348	0.054
Wheat_4	Transfer_ARR	YT_16_17‐YT_17_18	0.050	0.003	0.419	0.061
Wheat_4	RR	YT_17_18‐YT_16_17	0.048	0.004	0.334	0.099
Wheat_4	ARR	YT_17_18‐YT_16_17	0.044	0.003	0.412	0.104
Wheat_4	Transfer_RR	YT_17_18‐YT_16_17	0.044	0.003	0.417	0.102
Wheat_4	Transfer_ARR	YT_17_18‐YT_16_17	0.043	0.004	0.480	0.087

*Note*: NRMSE_SD denotes the standard deviation of NRMSE and Cor_SD denotes the standard deviation of Cor. In the transfer column, the notation “Env1‐Env2” denotes that Env1 is the environment used as a proxy and Env2 is the environment used as goal.

Abbreviations: ARR, analytic ridge regression; RR, ridge regression.

**TABLE A7:** Prediction performance in terms of normalized root mean square error (NRMSE) and Pearson's correlation (Cor) for the Wheat_5 dataset.

Dataset	Model	Transfer	NRMSE	NRMSE_SD	Cor	Cor_SD
Wheat_5	RR	YT_17_18‐YT_18_19	0.064	0.005	0.300	0.055
Wheat_5	ARR	YT_17_18‐YT_18_19	0.059	0.004	0.427	0.074
Wheat_5	Transfer_RR	YT_17_18‐YT_18_19	0.059	0.004	0.430	0.073
Wheat_5	Transfer_ARR	YT_17_18‐YT_18_19	0.055	0.003	0.529	0.068
Wheat_5	RR	YT_18_19‐YT_17_18	0.042	0.004	0.394	0.081
Wheat_5	ARR	YT_18_19‐YT_17_18	0.040	0.004	0.421	0.056
Wheat_5	Transfer_RR	YT_18_19‐YT_17_18	0.040	0.003	0.434	0.053
Wheat_5	Transfer_ARR	YT_18_19‐YT_17_18	0.037	0.003	0.562	0.044

*Note*: NRMSE_SD denotes the standard deviation of NRMSE and Cor_SD denotes the standard deviation of Cor. In the transfer column, the notation “Env1‐Env2” denotes the fact that Env1 is the environment used as a proxy and Env2 is the environment used as goal.

Abbreviations: ARR, analytic ridge regression; RR, ridge regression.
